# Ocular Manifestations in Head and Neck Cancer: A Cross-Sectional Study from a Tertiary Care Centre from South India

**DOI:** 10.22336/rjo.2023.56

**Published:** 2023

**Authors:** Thanmayashree Sathyanarayana, Chinmayee J Thrishulamurthy, Jasleen Kaur, Vanagondi Aishwarya Prakash, Kusuma Maddarahalli Jagadeesh, H Shafeeq Ahmed

**Affiliations:** * Department of Ophthalmology, Bangalore Medical College and Research Institute, India

**Keywords:** head and neck neoplasms, ocular manifestations, cross-sectional studies, visual acuity, early detection of cancer

## Abstract

**Introduction:** Head and neck cancers (HNCs) present a significant global health burden, especially in India, where oral cavity cancers, notably affecting the tongue, are prevalent. A substantial portion of global HNCs (57.5%) is concentrated in Asia, India contributing with 30%. Despite advancements, challenges persist due to HNCs’ invasive nature and metastatic potential. This study aims to explore the link between HNCs and ocular manifestations.

**Methods:** A cross-sectional study was conducted at Bangalore Medical College and Research Institute involving 47 patients with diagnosed HNCs and ocular complaints. Clinical evaluations encompassed visual acuity, anterior and posterior segment examinations, and specialized investigations when necessary.

**Results:** A diverse range of malignancies were observed, with SCC maxilla and xeroderma pigmentosa, each accounting for 10.63% of cases. Ocular examinations unveiled visual acuity challenges, anterior segment findings like masses, exotropia, pigmented lesions, and varied fundus abnormalities. The anterior segment findings encompassed masses often accompanied by protrusion or relative afferent pupillary defect (RAPD). Additionally, exotropia, pigmented lesions, and other conditions were observed. Fundus examination revealed a spectrum of findings, including media haziness (10.63%), lack of view (17.02%), and pale discs (6.38%). Treatment plans were diverse, including excision biopsies (42.55%), exenteration procedures, Mitomycin-C applications, and referrals for chemotherapy and radiotherapy.

**Conclusion:** The present study underscores the significance of ophthalmological assessment and investigations in patients with diagnosed HNCs, emphasizing the value of early detection and intervention.

**Abbreviations:** HNC = Head and Neck Cancer, OCT = Optical Coherence Tomography, WNL = Within Normal Limits, SCC = Squamous Cell Carcinoma, MRI = Magnetic Resonance Imaging, CT = Computed Tomography, RAPD = Relative Afferent Pupillary Defect, XP = Xeroderma Pigmentosa

## Introduction

Head and neck cancers (HNCs) pose a substantial global health burden, presenting challenges in terms of both morbidity and mortality, despite advancements in early detection and treatment strategies. Among the spectrum of anatomical sites susceptible to HNCs, oral cavity cancers, particularly those affecting the tongue, are prevalent in India [**1**]. Notably, 57.5% of worldwide head and neck cancer cases are concentrated in Asia, with India contributing significantly, accounting for 30% of all cancer instances [**2**,**3**]. In the Indian context, HNCs represent a considerable proportion of total cancer cases, comprising 29.8-50.4% of male cancer cases and 11-22% of female cancer cases [**4**].

The complexities linked to HNCs are heightened by their inclination towards local invasiveness and potential metastasis. These attributes emphasize the urgency for a holistic comprehension of the disease and the implementation of proficient management strategies [**5**].

Unfortunately, HNCs are frequently identified at advanced stages of tumor advancement [**6**]. This delay in detection can be attributed to the ambiguous and nonspecific nature of HNC symptoms, often leading individuals to dismiss them as transient ailments.

Of particular interest is the intricate relationship between HNCs and ocular manifestations, given the proximity of the eye to the head and neck region. Reports from clinical surveys indicate that ophthalmic involvement in metastatic malignancies can vary, with an incidence ranging from 0.7% to 4.7% [**7**]. Ocular complications may arise through various mechanisms, including hematogenous spread, direct invasion, raised intracranial pressure, and paraneoplastic phenomena. Additionally, therapeutic interventions such as surgery, radiation therapy, and chemotherapy tailored to tumor types can influence ocular outcomes [**8**]. 

The current study aims to quantify the occurrence and patterns of ophthalmic manifestations in patients diagnosed with head and neck malignancies.

## Methods

*Study Design*

This study employed a hospital-based cross-sectional design to comprehensively explore the relationship between head and neck malignancies and ocular manifestations. The research was conducted within the Department of Ophthalmology at Bangalore Medical College and Research Institute, India. The study was approved by the Institutional Ethics Committee and followed the tenets outlined in the 1964 Declaration of Helsinki.

*Data Collection*

A total of 47 patients diagnosed with confirmed cases of head and neck malignancies were included in this study. These patients, presenting with eye-related complaints, were referred from Kidwai Hospital between February 2021 to August 2022. Data collection was meticulously performed using a predesigned proforma that captured essential details of each participant’s medical history and ocular complaints.

*Clinical Evaluation*

All participants underwent a comprehensive clinical examination, conducted by trained ophthalmological professionals. This examination encompassed visual acuity assessment for both distance and near vision. Additionally, the anterior and posterior segments of the eye were thoroughly evaluated using slit lamp microscopy and direct/indirect ophthalmoscopy, respectively. 

For participants for whom further investigations were warranted, a range of specialized diagnostic procedures was undertaken. These investigations included B-scan ultrasonography, CT scan, MRI, fundus fluorescein angiography, and Optical Coherence Tomography (OCT). These procedures were performed with a keen focus on their clinical relevance and necessity for accurate diagnosis and treatment planning.

*Statistical Analysis*

The demographic and relevant clinical and pathological data of all these patients were tabulated on MS Excel and then analyzed.

## Results

**Table 1** provides a comprehensive insight into the sociodemographic and clinical profile of the study participants. The distribution of age groups revealed that the majority of participants (59.57%) fell into the category of individuals aged over 45 years. Gender distribution showed a higher representation of males (63.82%) compared to females (36.17%). Chief complaints varied among participants, with growth/swelling (65.95%), proptosis (65.95%), and diminution of vision (85.1%) emerging as the most frequent concerns. Smaller percentages reported other symptoms like inability to move with total ophthalmoplegia and burning sensation.

**Table 1:** Sociodemographic and clinical details of participants

PARAMETERS	RESULTS
Age	
15-30	11 (23.4%)
30-45	8 (17.02%)
>45	28 (59.57%)
Gender	
Male	30 (63.82%)
Female	17 (36.17%)
History of familial cancers	
Yes	1 (2.12%)
No	46 (97.87%)
Chief complaints	
Growth/Swelling	31 (65.95%)
Proptosis	31 (65.95%)
Diminution of vision	40 (85.1%)
Inability to move with total ophthalmoplegia	1 (2.12%)
Burning sensation	1 (2.12%)
General physical examination	
Skin freckling with ulcerative lesions around the nose	5 (10.63%)
Lymph node enlargement	10 (21.27%)
WNL	30 (63.82%)
Systemic examination	
High-grade fever	1 (2.12%)
Lump in the breast	2 (4.25%)
WNL	44 (93.61%)
WNL = Within Normal Limits	

**Table 2** presents a diverse spectrum of diagnoses among participants, encompassing various malignancies like adenocarcinomas, adenoid cystic carcinoma, Ewing’s sarcoma, glioblastoma multiforme, and squamous cell carcinomas (SCC) affecting different sites. Notably, SCC maxilla and xeroderma pigmentosa each constituted 10.63% of cases. Additionally, lymphoma, malignant melanoma, recurrent dermatofibrosis, and other conditions contributed to the complex landscape of head and neck malignancies.

**Table 2:** Diagnosis

DIAGNOSIS	RESULTS
Acute leukaemia	3 (6.38%)
Adenocarcinoma of breast	3 (6.38%)
Adenocarcinoma of sinus	1 (2.12%)
Adenoid cystic carcinoma of the maxilla	1 (2.12%)
Adenoid cystic carcinoma of the mandible	1 (2.12%)
Ewings sarcoma	2 (4.25%)
Follicular carcinoma of the thyroid	1 (2.12%)
Glioblastoma multiforme	1 (2.12%)
Lymphoma	5 (10.63%)
Malignant melanoma	1 (2.12%)
Mesenchymal tumor of maxilla	1 (2.12%)
Ovarian carcinoma	1 (2.12%)
Papillary carcinoma of the thyroid	1 (2.12%)
Parotid gland adenocarcinoma	2 (4.25%)
Recurrent dermatofibrosis	1 (2.12%)
SCC buccal mucosa	1 (2.12%)
SCC mandible	2 (4.25%)
SCC maxilla	5 (10.63%)
SCC nose	2 (4.25%)
SCC oesophagus	1 (2.12%)
SCC scalp	1 (2.12%)
Sella Turcica tumor	1 (2.12%)
Spend wing meningioma	2 (4.25%)
Xeroderma Pigmentosa	5 (10.63%)
SCC = Squamous Cell Carcinoma	

**Table 3** provides a comprehensive view of the ocular examination outcomes among participants. Notable observations included prevalent visual acuity challenges, such as counting fingers at varying distances (36.17%) and hand movements (42.55%). Diverse anterior segment findings ranged from masses often accompanied by protrusion or RAPD (Relative Anterior Pupillary Defect), to conditions like exotropia and pigmented lesions. Fundus examination revealed varied findings, including media haziness (10.63%), lack of view (17.02%), and pale discs (6.38%). 

**Table 3:** Summary of ocular examination findings and distribution in the patient population

OCULAR EXAMINATION	RESULTS
VISUAL ACUITY	
Counting fingers at 1 to 5 meters	17 (36.17%)
Counting fingers to hand movements	20 (42.55%)
Perception of light	8 (17.02%)
WNL	2 (4.25%)
ANTERIOR SEGMENT	
Anterior retinopathy	1 (2.12%)
Dry eye	1 (2.12%)
Exotropia	1 (2.12%)
LR palsy	1 (2.12%)
Mass	12 (25.53%)
Mass + Protrusion	18 (38.29%)
Mass + RAPD	1 (2.12%)
Panuveitis	1 (2.12%)
Pigmented lesion	1 (2.12%)
Protrusion	2 (4.25%)
RAPD	3 (6.38%)
RAPD + Exposure Keratopathy	2 (4.25%)
Total ophthalmoplegia	1 (2.12%)
WNL	2 (4.25%)
EXTRA-OCULAR MUSCLE MOVEMENTS	
Restriction	33 (70.21%)
WNL	14 (29.78%)
FUNDUS	
Anaemic retinopathy	2 (4.25%)
Choroidal folds	2 (4.25%)
Choroidal mass	1 (2.12%)
Disc edema	1 (2.12%)
Media hazy	5 (10.63%)
No view	8 (17.02%)
Pale disc	3 (6.38%)
WNL	25 (53.19%)
WNL = Within Normal Limits, RAPD = Relative Afferent Pupillary Defect	

**Table 4:** Treatment plan results and distribution in the patient population

TREATMENT PLAN	RESULTS
Biopsy	
Excision biopsy	20 (42.55%)
Incision biopsy	8 (17.02%)
Not done	2 (4.25%)
Treatment	
Antibiotic steroid	1 (2.12%)
Enucleation	1 (2.12%)
Exenteration	3 (6.38%)
Exenteration with mandibulectomy with lymph node dissection	1 (2.12%)
Exenteration with parotidectomy with lymph node dissection	1 (2.12%)
Exenteration with radiotherapy and chemotherapy	2 (4.25%)
Exenteration with total maxillectomy	16 (34.04%)
Exenteration with total parotidectomy with neck dissection	1 (2.12%)
Excision biopsy with margin clearance	1 (2.12%)
Exposure keratopathy treatment with lateral tarsorrhaphy	2 (4.25%)
Local excision with orbital exenteration	1 (2.12%)
Mitomycin-C	5 (10.63%)
Referred for chemotherapy	5 (10.63%)
Referred for radiotherapy	2 (4.25%)

**Table 4** presents the treatment plans undertaken for the study participants, revealing a diverse array of approaches. Predominantly, excision biopsy (42.55%) and various exenteration procedures, notably exenteration with total maxillectomy (34.04%), were prominent. A spectrum of treatments, including referrals for chemotherapy (10.63%) and radiotherapy (4.25%), as well as the application of Mitomycin-C (10.63%), were applied. 

## Discussion

The study discussed the interplay between head and neck malignancies and ocular manifestations, shedding light on critical insights that hold implications for both clinical practice and public health initiatives. The findings underscored the significance of age distribution, revealing a dominant representation of individuals above the age of 45 (59.6%). This observation prompts considerations for targeted screening campaigns and early detection strategies tailored to this demographic [**9**]. Furthermore, gender distribution highlighted a substantial male representation (63.8%), reflecting established global trends in head and neck cancer incidence.

With regards to ocular examination findings, the prevalence of conditions such as diminished vision (38%) and growth/swelling (30%) underscored the clinical relevance of comprehensive ophthalmic assessments in conjunction with head and neck cancer diagnosis. This finding offered a strong basis for integrating ocular evaluations into the diagnostic process, thereby enabling early interventions and improved patient outcomes. Regarding the diagnosis, the diverse range of malignancies identified among participants reinforced the need for personalized treatment strategies that aligned with the specific attributes of each malignancy. This underscored the role of multidisciplinary collaboration in providing holistic patient care.

It is important to note that patients with SCCs in multiple anatomical sites such as the maxilla, mandible, nose, scalp, and esophagus were identified to have ophthalmological manifestations in our study. SCCs are known to have a prevalence of 3-9% among HNCs [**10**]. Furthermore, given the SCCs’ invasive nature and tendency to metastasize, early ophthalmological assessments become pivotal in detecting any ocular manifestations. Regular eye evaluations can aid in both patient care and a comprehensive understanding of the SCCs’ impact on overall health.

Ophthalmological manifestations in XP (Xeroderma Pigmentosa) hold significant clinical importance due to the potential impact on both vision and overall patient management. The ocular manifestations in XP can range from photophobia, dryness, and conjunctival injection to more severe conditions like pterygium and ocular surface malignancies [**11**]. The close association between XP and ocular abnormalities highlights the need for regular ophthalmic examinations as a crucial component of patient care [**12**].

The visual acuity assessment revealed a considerable proportion of patients experiencing compromised vision, with 36.17% counting fingers at 1 to 5 meters, and 42.55% exhibiting counting fingers to hand movements. Notably, 17.02% had limited perception of light, indicative of varying degrees of visual impairment. These findings underscored the significant impact of head and neck malignancies on visual function and highlighted the importance of early detection and intervention to mitigate visual deterioration as discussed by Brooks et al. [**11**]. The prominence of Mass and Mass + Protrusion (38.29%) highlighted the potential for space-occupying lesions to cause proptosis and other visual disturbances, warranting careful examination and timely management to prevent further complications [**13**].

The occurrence of ocular motility restrictions in 70.21% of patients underscored the impact of these malignancies on extraocular muscle function. The presence of restrictions can lead to double vision, impaired eye movements, and overall diminished ocular motility, significantly affecting the quality of life for these patients [**14**]. Additionally, the observation of media haziness (10.63%) and pale disc (6.38%) in the fundus examination, along with choroidal folds and choroidal mass, highlighted the spectrum of posterior segment involvement. These findings emphasized the need for comprehensive fundus evaluation in patients with head and neck malignancies to detect potential retinal and optic nerve abnormalities [**15**].

The diversity of treatment plans and interventions observed in our study highlighted the complex nature of managing head and neck malignancies and the necessity for personalized approaches tailored to each patient’s condition. Excision biopsy was the predominant diagnostic method employed (42.55%), indicating the importance of histological confirmation in guiding treatment decisions. Such biopsies facilitate accurate characterization of the malignancy and its aggressiveness, playing a pivotal role in devising suitable therapeutic strategies.

From a public health standpoint, the study results emphasized the importance of raising awareness about the varied treatment options available for head and neck malignancies. Public education campaigns can play a crucial role in helping patients and their families make informed decisions about treatment choices and potential outcomes. Moreover, the findings of the study underscored the need for ongoing research and development to refine treatment protocols, enhance therapeutic efficacy, and reduce the potential complications associated with these interventions.

The study presented a comprehensive evaluation of ocular manifestations in patients with head and neck malignancies, offering valuable insights into the prevalence and types of ocular complications associated with these cancers. However, the study’s single-facility design and relatively small sample size of 47 patients might limit the generalizability of findings. Additionally, the cross-sectional nature of the study restricted the establishment of causal relationships and temporal associations between variables. Moreover, retrospective data collection could introduce recall bias or incomplete documentation. Nevertheless, the study demonstrated strengths in its comprehensive approach, involving a multidisciplinary team of medical specialists and utilizing various diagnostic and treatment methods. This approach enhanced the study’s clinical relevance by addressing head and neck malignancies from multiple angles. By emphasizing the importance of early detection, personalized treatment plans, and multidisciplinary collaboration, the study provided insights that could contribute to improved patient care. Furthermore, the study’s focus on ocular manifestations underscored the relevance of raising awareness and advocating for comprehensive care for individuals with head and neck malignancies. Future long-term longitudinal studies could help to a better understanding and could confirm the association between HNCs and ocular manifestations.

## Conclusion

In conclusion, this hospital-based cross-sectional study shed light on the intricate relationship between head and neck malignancies and ocular manifestations, emphasizing the need for a multidisciplinary approach to patient care. The findings underscored the significant burden that these cancers pose not only on patients’ general health but also on their ocular well-being. The study’s comprehensive evaluation of ocular status and treatment options provided valuable insights into addressing the complex challenges posed by these malignancies. While limitations in sample size and design must be acknowledged, the study’s merits lie in its meticulous data collection and its potential to guide further investigations into the field. Ultimately, this study reinforces the importance of early detection, tailored treatment strategies, and ongoing collaboration among medical disciplines, aiming to improve the quality of life for patients facing head and neck malignancies while highlighting the crucial role of ophthalmology in their comprehensive care.

**Conflict of Interest Statement**

The authors declare no conflict of interest.

**Informed Consent and Human and Animal Rights Statement**

Informed consent was obtained from the individuals included in this study.

**Authorization for the use of human subjects**

Ethical approval: The research related to human use complied with all relevant national regulations and institutional policies, followed the tenets of the Helsinki Declaration, and was approved by the Institutional Ethics Committee of Bangalore Medical College and Research Institute, India. 

**Acknowledgments**

None.

**Sources of Funding**

None.

**Disclosures**

None.

**Data sharing Statement**

None.
